# Association Between Suicide and Physical Illness in Japan: An Analysis Using Statistical Adjustment Based on Regional Attributes

**DOI:** 10.7759/cureus.82192

**Published:** 2025-04-13

**Authors:** Akihito Ueda, Masayoshi Koinuma

**Affiliations:** 1 Department of Internal Medicine, Medical Corporation Toujinkai, Fujitate Hospital, Osaka, JPN; 2 Doctoral Program in Pharmaceutical Sciences, Graduate School of Pharmaceutical Sciences, Teikyo Heisei University, Tokyo, JPN; 3 Faculty of Pharmaceutical Sciences, Teikyo Heisei University, Tokyo, JPN

**Keywords:** cerebral infarction, diabetes, healthcare workers, older age, suicide prevention

## Abstract

Background: Suicide is a serious social problem in Japan, and suicides related to health problems are predominant. To date, no studies have considered and adjusted for suicide-associated factors based on regional attributes when examining the relationship between physical illness and suicide.

Objective: Using open data, we examined the relationship between the number of outpatient visits by disease and the number of suicides per prefecture, while adjusting for regional confounding factors.

Methods: We conducted stepwise multiple regression analysis on data from 47 Japanese prefectures, adjusting the number of suicides per prefecture for suicide-associated factors based on regional attributes, such as the proportion of older people, household assets, happiness level, and sunlight hours. We analyzed six diseases potentially associated with suicide: neoplasms, gastric/duodenal ulcers, liver disease, glomerular disease/renal failure, diabetes, and cerebral infarction.

Results: Suicide was significantly associated with cerebral infarction and diabetes (scaled estimates: 1.10 and 0.842; adjusted power: 83.2% and 85.7%, respectively). When analyzing without adjustment for regional attributes, no significant associations were found, highlighting the importance of controlling for confounding factors. The proportion of older people (estimate: 0.343, p = 0.002) and household assets (estimate: −0.138, p = 0.004) were identified as significant regional factors affecting suicide rates. These findings suggest that both clinical factors and socioeconomic conditions must be considered in suicide prevention efforts.

Conclusions: Our findings highlight the importance of monitoring patients with cerebral infarction and diabetes for suicide risk, while considering regional socioeconomic factors. Primary healthcare workers, who are available, accessible, knowledgeable, and committed to providing care, may play a crucial role in suicide prevention by paying particular attention to these patient populations. These results provide valuable insights for developing targeted suicide prevention strategies in clinical settings.

## Introduction

Suicide is one of the most serious social problems in Japan. The 2021 White Paper on Suicide Prevention [1] by the Ministry of Health, Labor, and Welfare of Japan reported suicide mortality rates (annual number of deaths per 1,000 people) in seven major developed countries as follows: 16.1 in Japan, 14.7 in the United States, 13.1 in France, 11.6 in Germany, 11.3 in Canada, 7.3 in the United Kingdom, and 6.5 in Italy. Suicide mortality rates are also reported as 26.9 in South Korea, ranking highest globally, followed by 23.5 in Lithuania and 19.1 in Slovenia, with Japan ranking seventh overall [1].

According to suicide statistics from the National Police Agency of Japan, the number of suicides in Japan remained above 30,000 for 14 years, from 1998 to 2011. However, the number fell below 30,000 in 2012 and has been trending downward since then. This decrease is attributed to various factors, including improved economic conditions, government-led suicide prevention initiatives, and increased awareness and support for mental health. Nevertheless, in 2020, suicide rates began to rise again. From 1978 to the present, suicide rates in Japan have never fallen below 20,000. The total number of suicides in 2020 was 21,081.

Most suicide cases have a diverse and complex set of causes and backgrounds and occur in the context of a chain of various events. Among these, suicides attributed to health problems account for the largest proportion, about half of all cases [1]. In official Japanese statistics, "health problems" encompass both mental health disorders (e.g., depression, schizophrenia) and physical illnesses (e.g., cancer, stroke, diabetes). While mental illnesses such as depression, alcoholism, and schizophrenia are well-established risk factors for suicide, various physical illnesses also significantly contribute to suicide risk. According to a 2009 review on suicide by Hawton et al. [2], the following physical illnesses have been reported to be associated with suicide: malignant neoplasms, human immunodeficiency virus/acquired immunodeficiency syndrome (HIV/AIDS) infection, Huntington's disease, multiple sclerosis, epilepsy, peptic ulcer disease, kidney disease, spinal cord injury, systemic lupus erythematosus, and chronic pain. Although this review was published in 2009, its findings have continued to be supported by subsequent studies. Therefore, we cite it here as a representative summary of disease categories associated with suicide risk.

The World Health Organization (WHO) has produced a guide to suicide prevention for primary healthcare workers [3], which states that "some types of physical illnesses are associated with an increased suicide rate." Specific diseases named in this guide include neurological disorders, epilepsy, spinal or head injuries, cerebral infarction, cancer, HIV/AIDS, and other chronic conditions such as diabetes and multiple sclerosis.

Regional differences in suicide mortality rates have also been reported in Japan [1]. Previous studies have identified several factors associated with these regional differences. Suicide rates generally increase with age [4]; thus, it has been reported that the proportion of community-dwelling older people [5,6] correlates positively with suicide. Household assets [6-9] have also been reported to be negatively correlated with suicide. Aihara et al. [6] reported a positive correlation between aging and suicide rates for both men and women, whereas they found a negative correlation between the amount of savings and suicide rate among men, suggesting an association between job-seeking rate and suicide rate among women. Motohashi et al. [9] showed that annual income and savings amount correlated negatively with the number of patients with mental illness and that savings amount correlated negatively with the suicide rate. Moreover, happiness [10] and duration of sunlight (hereafter referred to as sunlight hours) [8,11] correlate with suicide rates. Van Schaik et al. [10] reported that “low happiness” correlated positively with suicidal behavior. Suzuki et al. [8] reported that the suicide rate correlated negatively with sunlight hours in both men and women. Egashira et al. [11], however, reported a positive correlation between suicide rate and sunlight hours, since seasonal fluctuations in suicide rates are similar to annual fluctuations in sunlight hours.

While many reports have considered the relationship between suicide and physical illnesses, to our knowledge, few previous studies have considered suicide-associated factors based on regional attributes as confounding factors in this specific context. Our literature search did not identify studies with a similar methodological approach, although we acknowledge that this was not a systematic review. We considered that statistical adjustment for suicide-associated factors based on regional attributes, as confounding factors, is necessary.

This study aimed to investigate the relationship between outpatient visits for specific physical illnesses, such as diabetes, cerebral infarction, gastric ulcer, neoplasm, liver disease, and kidney disease, and suicide mortality rates across Japanese prefectures, while adjusting for regional attributes such as age distribution, household assets, happiness level, and sunlight exposure.

In Japan, the suicide rate, which had been declining for over a decade, showed a temporary increase in 2020, coinciding with societal shifts during the COVID-19 pandemic. This raised renewed interest in identifying contributing factors.

## Materials and methods

Data collection

The number of suicides per 100,000 people in each prefecture in Japan for the year 2020 was published by the Ministry of Health, Labor, and Welfare of Japan [1].

The suicide data represent crude rates (number of suicides per 100,000 population) as reported by the Ministry of Health, Labor, and Welfare of Japan. To account for potential bias due to differences in age structure across prefectures, we included the proportion of older people (percentage of the population aged ≥65 years) as a key confounding variable in our analyses. We then selected six diseases that could be extracted from the injury and disease classification codes, as indicated to be potentially associated with suicide, as listed in the review by Hawton et al. [2] and in the WHO guidelines [3]. Neoplasms were selected from the major category in the injury and disease classification code, while gastric ulcers and duodenal ulcers, liver disease, glomerular disease/renal tubulointerstitial disease and renal failure, diabetes, and cerebral infarction were selected from the intermediate category.

These diseases were selected based on two main criteria: (1) the availability of outpatient visit data in the RESAS database and (2) their relatively high frequency in the general population. Other physical illnesses mentioned in the literature, such as spinal cord injury, HIV/AIDS, or systemic lupus erythematosus, were excluded due to either limited availability of data in RESAS or insufficient sample sizes for meaningful statistical analysis.

These disease categories were selected because they aligned with the conditions mentioned in the literature as having potential links to suicide risk and were clearly definable within the classification system used in the Regional Economy and Society Analyzing System (RESAS). The RESAS system has its own classification method, which is based on, but not identical to, the International Classification of Diseases codes. We then used the disease-specific number of outpatient visits for these diseases per 100,000 people in each prefecture, as recorded in RESAS, which is operated by the Cabinet Office and the Ministry of Economy, Trade, and Industry of Japan.

The RESAS disease classification system is derived from, but not identical to, the International Classification of Diseases (ICD) codes. Disease categories in this study were selected from the RESAS intermediate or major categories that most closely correspond to ICD-10 groupings. For example, "cerebral infarction" in RESAS corresponds broadly to ICD-10 code I63. However, because RESAS does not offer detailed ICD mapping, some level of approximation was required in disease selection.

Suicide-associated factors based on regional attributes (confounding factors)

The proportion of older people, household assets, happiness level, and sunlight hours were included as suicide-associated factors based on regional attributes that have been reported in previous research.

The “aging rate” (proportion of people aged ≥65 years in the total population) in each prefecture was evaluated based on the 2020 “Population Estimates” by the Ministry of Internal Affairs and Communications of Japan to define the proportion of older people [12]. Total household assets (per household) in each prefecture were evaluated based on the 2019 National Household Structure Survey by the Statistics Bureau, Ministry of Internal Affairs and Communications of Japan, to define household assets [13]. Happiness level in each prefecture was assessed based on the Second Regional (Prefectural) Sustainable Development Goals Survey 2020 [14]. “Annual sunlight hours” in each prefecture in 2020 were evaluated based on past meteorological data released by the Japan Meteorological Agency to define the amount of sunlight [15]. Data were aggregated by prefecture, yielding a sample size of 47.

Data analysis

A simple regression analysis was performed on suicide-associated factors based on regional attributes (proportion of older people, household assets, happiness level, and sunlight amount) to obtain summary statistics. Next, a simple regression analysis was performed using the number of suicides per 100,000 people as the objective variable and the proportion of older people, household assets, happiness level, and sunlight amount as the explanatory variables. A multiple regression model was created using factors that were found to be significantly correlated (p < 0.05) to estimate the adjusted suicide mortality rate per 100,000 people in each prefecture.

A simple regression analysis was then conducted using this adjusted suicide mortality rate as the objective variable and the number of outpatient visits by prefecture for the six diseases mentioned above as the explanatory variables. Highly relevant variables were judged as those with p < 0.05 and an effect size standard [16,17] of R² > 0.13 (effect size: medium). This threshold was selected based on Cohen's established criteria for effect sizes in behavioral and social science research [16]. A multiple regression analysis was performed on the diseases extracted as highly relevant variables using the forced entry method in order to create a model, with the aim of clarifying which diseases have the strongest impact on suicide. Next, a model was created using a stepwise method to improve the predictive ability of the model. The stopping rule in the stepwise method was set to the minimum Bayesian Information Criterion.

Thus, we first estimated the adjusted suicide mortality rate, then searched for suicide-associated diseases, and investigated suicide-associated diseases using unadjusted suicide mortality rates in order to assess the validity of this method (Appendices).

All statistical analyses were performed using JMP Pro version 17 (SAS Institute Inc., Cary, North Carolina). Multicollinearity was assessed using variance inflation factors (VIF), with all variables having VIF values below 1.20, indicating no concern. However, formal statistical tests for the normality and homoscedasticity of residuals were not conducted. This limitation was taken into account in the interpretation of the results.

Ethical considerations

This study exclusively utilized publicly available aggregated data at the prefectural level in Japan. No individual patient information was accessed or analyzed in this research. As such, individual informed consent was not required. Furthermore, due to the nature of the data used (publicly available aggregated statistics), this study did not require approval from an ethics committee.

All data were obtained from official government sources and public databases. The researchers adhered to all relevant guidelines for the ethical use of public data throughout the study.

## Results

A simple regression analysis was used to estimate adjusted suicide mortality rates, which identified the proportion of older people and household asset distribution as significantly associated factors. The results showed that prefectures with a higher proportion of older people (estimated value = 0.343, p = 0.0019) and fewer household assets (estimated value = −0.138, p = 0.0036) were significantly associated with suicide (Table 1).

**Table 1:** Estimation of the adjusted suicide mortality rate 95% CI: confidence interval indicating the 95% confidence range for estimated values. R^2^ value = 0.229, F-value = 6.539, p-value = 0.003 (multiple regression analysis).

Variable	Category	Estimated value	Scaled estimated value	Standard error	95% CI (lower, upper)	t-value	p-value	Analysis type
Proportion of older people (unit: %)	Intersection	7.822		2.932	(2.075, 13.569)	2.67	0.011	Univariate analysis
	Proportion of older people	0.343		0.104	(0.139, 0.547)	3.29	0.002	
	Intersection		17.427	0.283	(16.872, 17.982)	61.6	<0.001	Multiple regression analysis
	Proportion of older people		1.637	0.916	(–0.158, 3.432)	1.79	0.081	
Household assets (unit: million yen)	Intersection	20.640		1.086	(18.511, 22.769)	19.0	<0.001	Univariate analysis
	Household assets	–0.138		0.045	(–0.226, –0.050)	–3.07	0.004	
	Intersection		17.427	0.283	(16.872, 17.982)	61.6	<0.001	Multiple regression analysis
	Household assets		–1.275	0.902	(–3.043, 0.493)	–1.41	0.164	
Sunlight hours (unit: days)	Intersection	21.878		2.750	(16.488, 27.268)	7.95	<0.001	Univariate analysis
	Sunlight hours	–0.054		0.033	(–0.119, 0.011)	–1.63	0.110	
Happiness level (unit: points)	Intersection	30.928		9.056	(13.178, 48.678)	3.41	0.001	Univariate analysis
	Happiness level	–0.202		0.135	(–0.467, 0.063)	–1.49	0.143	

These two items were used to create a multiple regression model (Table 1) to estimate the predicted number of suicides per 100,000 people in each prefecture, which was then set as the adjusted suicide mortality rate. Next, a simple regression analysis was performed using the adjusted suicide mortality rates to identify suicide-associated diseases. Prefectures with a higher number of patients with diabetes (estimated value = 0.016, p = 0.001), cerebral infarction (estimated value = 0.027, p = 0.001), gastric ulcers and duodenal ulcers (estimated value = 0.124, p = 0.008), and neoplasms (estimated value = 0.014, p = 0.008) per 100,000 people tended to have a higher number of suicides (Table 2).

**Table 2:** Relationship between adjusted suicide mortality rate and suicide-associated diseases (univariate analysis)

Variable	Category	Estimated value	Standard error	95% CI (lower, upper)	t-value	p-value	R^2^ value
Diabetes	Intersection	14.548	0.816	(12.949, 16.147)	17.83	<0.001	
	Diabetes	0.016	0.004	(0.008, 0.024)	3.58	0.001	0.222
Cerebral infarction	Intersection	16.146	0.393	(15.376, 16.916)	41.10	<0.001	
	Cerebral infarction	0.027	0.008	(0.011, 0.043)	3.47	0.001	0.211
Gastric ulcer and duodenal ulcer	Intersection	16.152	0.460	(15.250, 17.054)	35.09	<0.001	
	Gastric ulcer and duodenal ulcer	0.124	0.044	(0.038, 0.210)	2.80	0.008	0.164
Neoplasm	Intersection	14.717	0.991	(12.775, 16.659)	14.85	<0.001	
	Neoplasm	0.014	0.005	(0.004, 0.024)	2.76	0.008	0.145
Liver disease	Intersection	16.882	0.393	(16.112, 17.652)	42.95	<0.001	
	Liver disease	0.027	0.018	(-0.008, 0.062)	1.50	0.141	0.048
Glomerular disease/renal tubulointerstitial disease and renal failure	Intersection	17.027	0.365	(16.312, 17.742)	46.69	<0.001	
	Glomerular disease/renal tubulointerstitial disease and renal failure	0.004	0.003	(-0.002, 0.010)	1.21	0.234	0.031

Creating a multiple regression model using the forced entry method with four diseases (neoplasms, gastric ulcers and duodenal ulcers, diabetes, and cerebral infarction) as factors showed that diabetes (scaled estimated value = 0.721, p = 0.014) and cerebral infarction (scaled estimated value = 0.876, p = 0.025) were significantly associated (Table 3).

**Table 3:** Relationship between the adjusted suicide mortality rate and suicide-associated diseases For multiple regression analysis, R^2^ value = 0.430, F-value = 6.987, p-value < 0.001. For stepwise method, R^2^ value = 0.370, F-value = 12.936, p-value <0.001.

Variable	Scaled estimated value	Standard error	95% CI (lower, upper)	t-value	p-value	Adjusted power	Analysis type
Intersection	17.368	0.131	(17.106, 17.630)	132.38	<0.001	1.000	Multiple regression analysis
Diabetes	0.721	0.279	(0.163, 1.279)	2.58	0.014	0.612	
Cerebral infarction	0.876	0.376	(0.124, 1.628)	2.33	0.025	0.510	
Gastric ulcer and duodenal ulcer	0.471	0.290	(-0.109, 1.051)	1.62	0.113	0.221	
Neoplasm	0.243	0.321	(-0.399, 0.885)	0.76	0.453	0.050	
Intersection	17.427	0.122	(17.183, 17.671)	142.30	<0.001	1.000	Stepwise method
Diabetes	0.842	0.253	(0.344, 1.340)	3.33	0.002	0.857	
Cerebral infarction	1.101	0.342	(0.430, 1.772)	3.22	0.002	0.832	

The R² value of the model was 0.430, and the p-value of the analysis of variance was <0.001. The variance inflation factor, which is an indicator of multicollinearity, was less than 1.20 for all factors, and no multicollinearity was observed.

Next, the results of creating a multiple regression model using a stepwise method showed that diabetes and cerebral infarction remained as significant factors. The scaled estimated values were 0.842 and 1.101, respectively, and their p-values were 0.002 and 0.002, respectively. Additionally, the values for the adjusted power were 85.7% and 83.2%, respectively (Table 3).

Furthermore, when we searched for suicide-associated diseases using unadjusted suicide mortality rates in order to examine the validity of this method, no significant factors met the conditions for simple regression analysis among all the diseases.

## Discussion

Principal results

Several studies have been conducted on the relationship between suicide and physical illness, but most of these studies involved analyses focused on a single disease and did not include adjustment for confounding variables. However, in the present study, we simultaneously adjusted for confounding variables and evaluated the relationship between multiple diseases and suicide, which has not been reported previously. Using open regional attribute data, we analyzed six diseases indicated as potentially associated with suicide (neoplasms, gastric ulcers and duodenal ulcers, liver disease, glomerular disease/renal tubulointerstitial disease, renal failure, diabetes, and cerebral infarction) by adjusting for suicide-associated factors based on regional attributes, such as the proportion of older people and household assets. We found that diabetes and cerebral infarction were associated with suicide mortality rates. In contrast, the unadjusted analysis failed to reveal any significant correlations, underscoring the necessity of adjusting for regional attributes to clarify the relationship between suicide and disease. Therefore, our methodological approach demonstrates that conducting an analysis based on regional attributes is essential for accurately identifying suicide-associated diseases. In this study, we showed specifically that diabetes and cerebral infarction were associated with suicide by analyzing open regional attribute data while adjusting for regional confounding factors.

Comparison with prior work

Data related to public health have increasingly become publicly available in recent years. A rich array of socioeconomic data, such as those on the role of public services, including medical care, can be obtained on a prefecture basis, allowing regional analysis in Japan. The ability to use such open data facilitates analyses such as the present study. In addition, since this was an ecological study using aggregated prefectural-level data, individual-level causal relationships cannot be inferred. The potential for ecological fallacy should be acknowledged when interpreting these findings, and further research using individual-level data is warranted.

Judging from the scaled estimated values obtained from our multiple regression analysis results, we clarified that cerebral infarction was the disease most strongly associated with suicide. It has been reported that the risk of suicide is significantly higher for the five-year period after the onset of cerebral infarction (risk ratio = 10.2) [18]. Yamauchi et al. reported that the reason for the increased risk of suicide after cerebral infarction onset was due to the increased risk of depression, which they considered to be a risk factor for suicide. The significant correlation between suicidal ideation and depression after the onset of cerebral infarction was also indicated in a meta-analysis by Zhang et al. (odds ratio = 2.50) [19]. Activity limitation due to physical illness has been reported to increase the risk of suicide (odds ratio = 3.13) [20]. Cerebral infarction may cause activity limitation due to physical disability and other sequelae. This activity limitation may be a trigger for suicide.

Diabetes was found to be the second most strongly suicide-associated disease after cerebral infarction onset. Sher conducted a meta-analysis of studies from many countries that investigated suicide risk in patients with diabetes and reported that diabetes increases the risk of suicide by 1.6 times [21]. Additionally, a report from Japan [22] stated that “diabetes increases suicide risk by 3.53 times.” Furthermore, a review paper by Conti et al. [23] reported that “Diabetes was found to be significantly associated with a marked increase in suicidal behaviors and suicidal ideation, especially in patients with depressive symptoms.” Diabetes causes various activity limitations due to complications, and these activity limitations may be a trigger for suicide [20].

For gastric ulcers and duodenal ulcers, the forced entry multiple regression model did not reach statistical significance (p = 0.11). However, Knop et al. previously reported that 13.7% of deaths in patients who underwent surgical treatment for duodenal ulcers were due to suicide, and 50% of the suicide cases were due to alcoholism [24]. A relationship between peptic ulcers and stress has long been known. A recent report on the incidence of peptic ulcers before and after the Great East Japan Earthquake [25] revealed that the incidence of peptic ulcers increased by 1.5 times after the earthquake, suggesting that psychosocial stress induced peptic ulcers and subsequently suicide.

We did not identify a significant association between neoplasms and suicide rates. However, this finding should be interpreted cautiously and in the context of existing literature. Previous studies have consistently demonstrated a relationship between cancer and suicide risk. For instance, Anguiano et al. conducted a literature review of 73 peer-reviewed studies and revealed that suicide risk is higher in patients with cancer than in the general population [26]. Additionally, Calati et al. stated that “cancer diagnosis is associated with increased suicide” [27].

The discrepancy in the association between neoplasms and suicide risk found in our study compared with that in previous research requires further examination. The discrepancy between our study and these previous studies [26,27] might be attributed to several factors. First, we used aggregated data at the prefectural level, which may have obscured individual-level associations. In contrast, previous studies involved individual-level data, allowing the researchers to detect more nuanced relationships between cancer diagnosis and suicide risk. Second, we analyzed neoplasms as a single category, whereas the impact on suicide risk may vary significantly depending on the type and location of the tumor. For example, Hu et al. reported higher suicide risks for cancer types with a poor prognosis and high symptom burden in the first two years after diagnosis, including oral cavity and pharynx, esophagus, stomach, brain and other nervous system, pancreas, and lung cancers [28].

We used the results of this study as a basis to consider the type of behavior that primary healthcare workers should exhibit as guardians against suicide. The majority of suicide victims are thought to suffer from some type of diagnosable mental illness [3]. Even for cerebral infarction and diabetes, which appear to have a particularly strong impact on suicide mortality rates, previous research has indicated that the risk of suicide may increase in patients with depressive symptoms [18,19,23]. Health systems have traditionally focused on suicide prevention for patients with diagnosed mental illness or those expressing suicidal ideation. However, this targeted approach has limited effectiveness, as most people who die by suicide do not have a diagnosed mental illness or receive specialized behavioral health treatment. Ahmedani et al. reported that 83% of people who died by suicide in the United States had received medical care in the year prior to death, but only 45% had a diagnosis for their mental health disorder or substance use, 14% received mental health-related inpatient treatment, and 29% had received specialized behavioral health outpatient treatment [29]. However, attempting to spread suicide prevention to all patients is burdensome and costly. In this study, the RESAS open data were used to identify clinical risk factors for suicide. This method may help to develop algorithms for more appropriately detecting suicide risk in clinical settings in future.

The WHO has developed a suicide prevention guide for primary healthcare workers [3]. For the question, "Why Focus on Primary HealthCare Staff?" the reason given was that "they are available, accessible, knowledgeable, and committed to providing care." The guide also stated that "primary care clinicians write more prescriptions for antidepressants than mental health clinicians and see patients more often in the month before their death by suicide" [30]. Primary care physicians should thus play a major role in preventing suicide, which remains a major public health problem.

Although the effect sizes observed in this study were statistically significant, their clinical significance should also be considered. The identified associations between suicide and diseases such as diabetes and cerebral infarction suggest potential targets for risk assessment in public health settings. However, the magnitude of the effect, while meaningful at the population level, may be modest in individual clinical contexts. These findings should thus be interpreted as supportive of prioritizing certain populations for suicide prevention strategies, rather than as definitive indicators of risk at the individual level.

Although the model demonstrated a moderate explanatory power (R² = 0.430), this also indicates that more than half of the variability in suicide mortality rates remains unexplained. This underscores the multifactorial nature of suicide and the need for further research to identify other contributing factors.

Moreover, the possibility of reverse causality should be considered. For example, depression may lead to poor disease management and exacerbate physical conditions such as diabetes or cerebral infarction, thereby increasing suicide risk.

In addition, since this was an ecological study using aggregated prefectural-level data, individual-level causal relationships cannot be inferred. The potential for ecological fallacy should be acknowledged when interpreting these findings, and further research using individual-level data is warranted.

Importance of adjusting for confounders

Our study's findings highlight the critical importance of adjusting for confounding factors in ecological studies. The stark contrast between our adjusted and unadjusted analyses, where significant associations between physical illnesses and suicide emerged only after controlling for regional attributes, demonstrates how powerful confounding variables can obscure meaningful relationships in population-level research. Without such adjustment, our analysis would have failed to identify the significant associations between cerebral infarction, diabetes, and suicide rates, potentially leading to erroneous conclusions about the relationship between physical illness and suicide.

Translating population-level findings to clinical practice

While our study identifies significant associations between certain physical illnesses and suicide rates at the prefectural level, translating these findings to individual clinical practice requires careful consideration. Population-level associations cannot directly predict individual risk without finer-grained data and validation in clinical cohorts. Nevertheless, our findings provide valuable direction for clinicians and healthcare systems.

Rather than attempting to implement a prediction model based on ecological data, a more appropriate clinical application would be heightened awareness and targeted screening. For instance, our findings suggest that primary care physicians and specialists who treat patients with diabetes or cerebral infarction should consider incorporating brief mental health assessments into routine care protocols for these populations. This might include regular screening for depression using validated tools such as the PHQ-9, or monitoring for suicidal ideation during follow-up appointments, particularly for patients with additional risk factors such as advanced age or socioeconomic challenges.

Healthcare systems could also use these findings to prioritize resources for integrated care models in regions with a high prevalence of these conditions. The implementation of collaborative care, where mental health professionals work alongside primary care providers, might be particularly beneficial for patients with diabetes and cerebral infarction. While our regional model cannot predict individual suicide risk with precision, it identifies patient populations that might benefit from enhanced mental health support within existing clinical frameworks.

Limitations

Our analysis is based on data from all 47 Japanese prefectures, which represents the complete set of prefectural administrative divisions in Japan rather than a sample. While this limited number of observations is an inherent constraint in prefecture-level analyses in Japan, it may affect the statistical power of our analyses, particularly for detecting smaller effect sizes. Our use of BIC as a stopping criterion in the stepwise regression and confirmation of low multicollinearity (VIF < 1.20) helped mitigate potential issues related to the limited number of observations. Alternative approaches using different units of analysis (such as smaller administrative divisions) would introduce different methodological challenges and would not necessarily preserve the important regional socioeconomic factors that were central to our study design.

A limitation of this study was that we analyzed the number of patients who had been diagnosed with a disease in some way, and not the actual number of patients in each prefecture, including those who had not been examined. In other words, it is not possible to take into account patients not captured in the data whose diseases have not been diagnosed through medical examination. Furthermore, the data used in this study were specific to each prefecture. Therefore, it is not possible to retrieve information such as when a single patient is examined for multiple diseases, and it is also not possible to take into account the severity of each disease in each patient. However, the statistical power for the obtained results was over 80%, and hence the results of the analysis were considered to be satisfactory.

In addition, this study was limited by the lack of data on individual-level psychosocial and psychiatric factors that are known to be associated with suicide risk, such as previous suicide attempts, depression, bipolar disorder, schizophrenia, alcohol or substance use, medication side effects, unemployment, and social isolation. These variables were not available in the publicly accessible, prefectural-level data used in this analysis. As with all ecological studies, our findings are subject to the risk of ecological fallacy: the associations we observed at the prefectural level cannot be directly translated to individual-level causation without supporting individual-level data. Additionally, the cross-sectional nature of our study prevents us from determining the direction of causality. There is a possibility of reverse causality, where underlying mental health conditions might simultaneously lead to poor disease management (potentially increasing the prevalence of conditions like diabetes) and elevated suicide risk, rather than the physical conditions themselves directly increasing suicide risk.

Additionally, we did not perform formal diagnostic tests for the normality or homoscedasticity of residuals in the regression models, which may affect the robustness of the statistical inference. As a result, residual confounding may exist and should be considered when interpreting the findings. We also acknowledge that the use of stepwise regression may increase the risk of overfitting, especially given the limited sample size (n = 47). In this study, stepwise selection was used as an exploratory tool to improve model parsimony, and results should be interpreted with caution.

Moreover, several important factors such as the prevalence of mental illness, unemployment rate, and alcohol consumption were not included due to the lack of reliable, publicly available data at the prefectural level. As such, these unmeasured confounders may have influenced the results and should be considered in the interpretation of findings.

The suicide data used in this study were from the year 2020, during which the COVID-19 pandemic and related societal changes occurred. However, the potential influence of the pandemic, including changes in social behavior, economic conditions, or health service access, was not specifically adjusted for in this analysis.

## Conclusions

We analyzed the number of suicides in each prefecture by adjusting for suicide-associated factors based on known regional attributes, such as the proportion of older people and household assets. Cerebral infarction and diabetes were strongly associated with suicide. These significant associations underscore the importance of considering these clinical risk factors in suicide prevention efforts. We hope that the findings obtained through this study will be utilized to assist with suicide prevention among patients with whom primary healthcare workers interact. Moreover, the integration of open data, such as the RESAS used in this study, may contribute to the development of algorithms that allow more accurate detection of suicide risk in clinical settings.

## Figures and Tables

**Figure 1:**
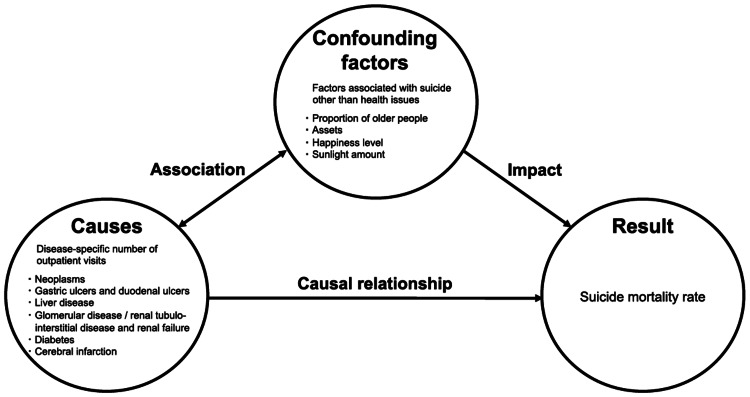
Conceptual Model of Disease-Specific Outpatient Visits and Suicide Mortality Rates

## References

[1] (2025). Ministry of Health, Labor, and Welfare of Japan. 2021 White paper on suicide prevention. https://www.mhlw.go.jp/stf/seisakunitsuite/bunya/hukushi_kaigo/seikatsuhogo/jisatsu/jisatsuhakusyo2021.html.

[2] Hawton K, van Heeringen K (2009). Suicide. Lancet.

[3] (2025). World Health Organization. Preventing suicide: a resource for primary health care workers. https://apps.who.int/iris/bitstream/handle/10665/67603/WHO_MNH_MBD_00.4.pdf?sequence=1&isAllowed=y.

[4] Shah A (2007). The relationship between suicide rates and age: an analysis of multinational data from the World Health Organization. Int Psychogeriatr.

[5] Tanaka N (1998). Relationship of suicide mortality to aging: modification of the aging effects by socioeconomic factors. J Kyorin Med Soc.

[6] Aihara H, Iki M (2003). An ecological study of the relations between the recent high suicide rates and economic and demographic factors in Japan. J Epidemiol.

[7] Suzuki T, Suka M, Yanagisawa H (2013). Analysis of trends on suicide mortality rates and regional factors in prefectures (Article in Japanese). J Health Welf Stat (Kosei no Shihyo).

[8] Miyoshi H, Sanada K (1993). Suicide mortality and economic fluctuation factors: Results of multiple regression analysis (Article in Japanese). J Jpn Ass Rural Med.

[9] Motohashi H, Fujimoto A, Sakane T, Yamamoto A, Yano Y (2013). Social factors of mental disorder and suicide in Japan-for understanding circumstance of suicides in each prefecture (Article in Japanese). Yakugaku Zasshi.

[10] van Schaik P, Peng Y, Ojelabi A, Ling J (2019). Explainable statistical learning in public health for policy development: the case of real-world suicide data. BMC Med Res Methodol.

[11] Egashira K, Suzuki T, Abe K (1986). Suicide seasonality and sunshine (Article in Japanese). Jpn J Biometeorol.

[12] (2025). Ministry of Internal Affairs and Communications of Japan. Population estimates 2020. https://www.e-stat.go.jp/stat-search/files.

[13] (2025). Statistics Bureau, Ministry of Internal Affairs and Communications of Japan. National household structure survey. https://www.stat.go.jp/data/zenkokukakei/2019/pdf/gaiyou0518.pdf.

[14] (2025). Regional SDGs and ESG Research Institute. The second regional (prefectural) SDGs survey. https://news.tiiki.jp/articles/4562.

[15] (2025). Manabi Info. Past weather data. https://manabi-info.com/japan-0116/.

[16] Asai T (2022). Statistics Note-25 effect size (1) (Article in Japanese). Jpn J Anesthesiol.

[17] Cohen J (1988). Statistical Power Analysis for the Behavioral Sciences. https://www.utstat.toronto.edu/brunner/oldclass/378f16/readings/CohenPower.pdf.

[18] Yamauchi T, Inagaki M, Yonemoto N (2014). Death by suicide and other externally caused injuries after stroke in Japan (1990-2010): the Japan Public Health Center-based prospective study. Psychosom Med.

[19] Zhang S, Wang A, Zhu W, Qiu Z, Zhang Z (2022). Meta-analysis of risk factors associated with suicidal ideation after stroke. Ann Gen Psychiatry.

[20] Onyeka IN, Maguire A, Ross E, O'Reilly D (2020). Does physical ill-health increase the risk of suicide? A census-based follow-up study of over 1 million people. Epidemiol Psychiatr Sci.

[21] Sher L (2022). Suicide in diabetes: an important but underappreciated problem. Mol Psychiatry.

[22] Fukunaga A, Hu H, Inoue Y (2020). Diabetes, prediabetes, and suicide deaths in a Japanese working population. J Psychosom Res.

[23] Conti C, Mennitto C, Di Francesco G, Fraticelli F, Vitacolonna E, Fulcheri M (2017). Clinical characteristics of diabetes mellitus and suicide risk. Front Psychiatry.

[24] Knop J, Fischer A (1981). Duodenal ulcer, suicide, psychopathology and alcoholism. Acta Psychiatr Scand.

[25] Kanno T, Iijima K, Abe Y (2013). Peptic ulcers after the Great East Japan earthquake and tsunami: possible existence of psychosocial stress ulcers in humans. J Gastroenterol.

[26] Anguiano L, Mayer DK, Piven ML, Rosenstein D (2012). A literature review of suicide in cancer patients. Cancer Nurs.

[27] Calati R, Filipponi C, Mansi W (2021). Cancer diagnosis and suicide outcomes: umbrella review and methodological considerations. J Affect Disord.

[28] Hu X, Ma J, Jemal A (2023). Suicide risk among individuals diagnosed with cancer in the US, 2000-2016. JAMA Netw Open.

[29] Ahmedani BK, Simon GE, Stewart C (2014). Health care contacts in the year before suicide death. J Gen Intern Med.

[30] McDowell AK, Lineberry TW, Bostwick JM (2011). Practical suicide-risk management for the busy primary care physician. Mayo Clin Proc.

